# An early warning indicator of mortality risk in patients with COVID-19: the neutrophil extracellular traps/neutrophilic segmented granulocyte ratio

**DOI:** 10.3389/fimmu.2024.1287132

**Published:** 2024-01-29

**Authors:** Qiong Wang, Yu Qin, Jingyun Ma, Kehao Zhou, Guiping Xia, Ya Li, Li Xie, Richmond Godwin Afful, Qian Lan, Xingyu Huo, Jian Zou, Hailin Yang

**Affiliations:** ^1^ The Affiliated Wuxi People’s Hospital of Nanjing Medical University, Wuxi People’s Hospital, Wuxi Medical Center, Nanjing Medical University, Wuxi, China; ^2^ Key Laboratory of Industrial Biotechnology, Ministry of Education, School of Biotechnology, Jiangnan University, Wuxi, China; ^3^ School of Internet of Things Engineering, Jiangnan University, Wuxi, China

**Keywords:** COVID-19, peripheral blood, smudge cells, neutrophil extracellular traps (NETs), NETs/neutrophilic segmented granulocyte ratio, thrombus

## Abstract

**Background:**

Neutrophil extracellular traps (NETs) play a key role in thrombus formation in patients with coronavirus disease 2019 (COVID-19). However, the existing detection and observation methods for NETs are limited in their ability to provide quantitative, convenient, and accurate descriptions of *in situ* NETs. Therefore, establishing a quantitative description of the relationship between NETs and thrombosis remains a challenge.

**Objective:**

We employed morphological observations of blood cells and statistical analyses to investigate the correlation between the NETs/neutrophilic segmented granulocyte ratio and mortality risk in patients with COVID-19.

**Methods:**

Peripheral blood samples were collected from 117 hospitalized patients with COVID-19 between November 2022 and February 2023, and various blood cell parameters were measured. Two types of smudge cells were observed in the blood and counted: lymphatic and neutral smudge cells. Statistical data analysis was used to establish COVID-19 mortality risk assessment indicators.

**Results:**

Morphological observations of neutrophilic smudge cells revealed swelling, eruption, and NETs formation in the neutrophil nuclei. Subsequently, the NETs/neutrophilic segmented granulocyte ratio (NNSR) was calculated. A high concentration of NETs poses a fatal risk for thrombus formation in patients. Statistical analysis indicated that a high NNSR was more suitable for evaluating the risk of death in patients with COVID-19 compared to elevated fibrinogen (FIB) and D-dimer (DD) levels.

**Conclusion:**

Observing blood cell morphology is an effective method for the detection of NETs, NNSR are important markers for revealing the mortality risk of patients with COVID-19.

## Introduction

In 2019, the COVID-19 pandemic caused by severe acute respiratory syndrome coronavirus 2 (SARS-CoV-2) virus was a significant public health event that affected the world (1, 2). Viral infections can cause death or severe cascading reactions such as cytokine storms, thrombosis, pulmonary embolism, myocardial infarction, or stroke (3, 4).

SARS-CoV-2 binds to the ACE-2 receptor on endothelial cells and induces the release of inflammatory cytokines. Activated endothelial cells produce monocyte chemoattractant factors, such as CCL2 and P-selectors, adhesion molecules, and tissue factor (TF) (5, 6). Circulating pathogen-associated molecular patterns (PAMPs), damage-associated molecular patterns (DAMP), and cytokines can activate blood monocytes, leading to the expression and release of monocyte-derived vesicles. The release of vesicles, activated monocytes, and TF expression in endothelial cells leads to the activation of the extrinsic coagulation pathway (7). Activated endothelial cells express P-selectin, which binds to P-selectin glycoprotein ligands (PSGL) on neutrophils, inducing neutrophil extracellular traps (NETs) and activating the intrinsic coagulation pathway (8). Additionally, endothelial cell activation causes a decrease in nitric oxide (NO) and an increase in von Willebrand factor (VWF), leading to vascular constriction, stasis, and platelet aggregation. Inhibition of the endogenous anticoagulation pathway, including tissue factor pathway inhibitors (TFPI), antithrombin, and protein C expression downregulation, is due to the release of plasminogen activator inhibitor 1 (PAI 1) (9). Activation of the intrinsic and extrinsic coagulation pathways and inhibition of the endogenous anticoagulation pathway can cause thrombus formation (10).

NETs play an important role in thrombus formation in patients with COVID-19, however, they are difficult to observe directly. Methods for detecting NETs include enzyme-linked immunosorbent assay (ELISA), immunofluorescence staining, fluorescence spectroscopy, flow cytometry (FCM), and electron microscopy (11). ELISA is sensitive, rapid, low-cost, and automated; however, it has poor reproducibility. Plasma DNA quantification requires centrifugation, which can interfere with other NETs components (12–14). Immunofluorescence staining with antibodies against NETs components and embedded DNA dyes has become the preferred method for qualitative and semi-quantitative detection of NETs; however, antibodies primarily target peroxidases and histones, which may be difficult to distinguish from other substances (15–17). Fluorescence spectroscopy allows high-throughput and rapid detection; however, not all cf-DNA originates from NETs (18, 19). FCM is a reliable method for analyzing thousands of cells in each sample, but it may focus on the detection of NETosis in progress and miss dissolved or late-stage NETosis cells as well as cit-H3-negative NETs (20–22). Electron microscopies, including scanning electron microscopy (SEM) and transmission electron microscopy (TEM), have become important tools for detecting NETs because of their visual characteristics, but may require verification by immunofluorescence microscopy (23). However, these methods are cumbersome and do not meet the requirements for rapid and convenient clinical use.

Neutral smudge cells have been reported in patients with COVID-19 (24). Smudge cells in the peripheral blood usually refer to lymphatic smudge cells; however, in our study, we found a large number of suspicious non-lymphatic smudge cells in the peripheral blood of patients with COVID-19, which requires further investigation.

## Materials and methods

### Human subjects

Demographic and clinical data were collected from 117 patients admitted to Wuxi People’s Hospital affiliated with Nanjing Medical University in China between November 2022 and February 2023. Among them, 107 had a confirmed diagnosis of COVID-19 and 10 were negative controls. Peripheral blood was collected from all patients and detailed information on age, sex, and medical history was recorded.

### Hematology assay *in vitro*

Elbow venous blood (2.5 mL) was drawn from all subjects. Blood cell parameters, including red blood cell parameters, white blood cell parameters, and platelet concentration (PLT) were directly determined using an automated hematology analyzer (Sysmex XN-20 (Schreiber and Farrar)).

### Hematology slide analysis

The automated hematology slide preparation system (Sysmex SP-10) automatically tested the slides if the patient’s routine blood test results were abnormal, triggering relevant review criteria. Images and data were analyzed using CellaVision software (RL-10191). Two independent skilled operators reviewed the blood smear images. Morphological analysis of smudge cells was independently performed by Jingyun Ma and Qiong Wang, a physician proficient in peripheral blood smear analysis. They have long been involved in the accurate identification of peripheral blood cell images using CellaVision software, and a review of related reports.

Analysis indicators of smudge cells morphology:

Smudge cells were divided into two categories.

1. Lymphatic smudge cells are round or round-like cells with no cytoplasm and degenerated nuclei. The nucleus appeared swollen, with a blurred structure and uniformly stained lavender.
2. Neutral smudge cells are uneven in size, lack a fixed shape, have reticular, lobulated nuclei that are depolymerized with granular characteristics, and are stained in a uniform lavender.

### Albumin smudge cell experiment

Bovine serum albumin (20%) was mixed with EDTA-K2 anticoagulated whole blood at a ratio of 1:5 (v/v), pushed onto a slide using an automated blood system (Sysmex SP-10) and stained with DI-60. CellaVision (RL-10191) was used for white blood cell classification and morphology analysis, and the results were compared with those of blood smears without albumin treatment.

### Definition of “mild, moderate, severe, and critical”

This definition is based on the “Diagnosis and Treatment Protocol for Novel Coronavirus Infection (trial version 10), issued by the People’s Republic of China.

### Definition of peripheral blood neutrophils count

The number of neutrophils in 100 white blood cells in peripheral blood smears was determined using CellaVision (RL-10191), excluding neutrophilic promyelocytes, neutrophilic myelocytes, neutrophilic metamyelocytes, and neutrophilic stab granulocytes, and counting only functionally mature segmented neutrophils.

### Definition of smudge cells and neutral smudge NETs count

Peripheral blood smudge cell count: The number of cells in the cell classification of peripheral blood smudges was counted using CellaVision (RL-10191).

Lymphatic smudge cell count: Peripheral blood was observed using CellaVision (RL-10191), and cells with only one degenerated nucleus, no cytoplasm, swollen nuclei, an unclear nuclear structure, and uniformly stained light-purple nuclei were observed.

Neutral smudge NETs count: Peripheral blood was observed using CellaVision (RL-10191), and cells with mesh-like segmented nuclei, degranulation, and granular features, defined as NETs, were counted. Features of non-neutrophil extracellular traps include bare nuclei, staining, and lymphocyte smears without filaments or granules.

### Definition of two parameters related to NETs

Definition of two parameters related to NETs: The number of neutrophil extracellular traps (NETs) in the peripheral blood (per 100 cells) refers to the number of neutrophil smudges counted by the CellaVision software in 100 white blood cells. The neutrophil extracellular trap-segmented neutrophil ratio (NNSR) refers to the ratio of the number of neutrophil smudges to the number of segmented neutrophils counted in 100 white blood cells using CellaVision software.

### Demarcation of thresholds and reference intervals

Logarithmic normal analysis was used to convert NNSR into the corresponding logarithm, and the normal distribution was presented after transformation (p<0.05, Shapiro–Wilk normal test). The mean ± 95% CI was used as the effective reference interval for each group. To mitigate false positives, NNSR is treated as suspected cases between the lower threshold mean, NNSR < lower thresholds, which are low-risk death patients, and NNSRs > mean value, for patients at high risk of death.

### Quantification and statistical analysis

Statistical analyses were performed using Origin 2020 and IBM SPSS Statistics version 27. To evaluate the significance of the count results, a non-paired t-test with Welch correction was applied when both sample groups passed the Shapiro–Wilk normality test. If one or more groups failed the normality test, a non-parametric multivariate analysis of variance was performed on the indicator diagram with multiple queues. Unless otherwise indicated, all P-values were adjusted for multiple comparisons. Statistical significance was defined as p < 0.05. Unless otherwise specified, error bars in all graphs represent one standard deviation.

## Results

### Compare the demographic, clinical, and laboratory characteristics of COVID-19 surviving and non-surviving cases

Patients with COVID-19 were classified into mild, moderate, severe, and critically ill categories, with 42 and 86 patients treated in the ICU and non-ICU settings, respectively. There were no significant differences in complications or underlying diseases among the patients. Cardiovascular diseases (76%) had the highest proportion, followed by diabetes and heart disease (33% and 22%, respectively), whereas kidney disease and other comorbidities were less common (16% and less than 7%, respectively) (**Table 1**).

**Table 1:** Patient characteristics.

Variable	Total (n=117)	Control(non-COVID-19) (n=10)	Mild- COVID-19 (n=19)	Moderate- COVID-19(n=47)	Severe- COVID-19 (n=15)	Especially severe- COVID-19 (n=12)	Dead(n=14)	*p*-value
Age, years	75(28-102)	72(41-86)	78(47-96)	74(45-91)	78(43-94)	79(28-102)	74(68-94)	0.0799
Sex								0.1528
Female	39(33.3%)	6(60.0%)	8(42.1%)	16(34.0%)	4(26.7%)	3(25.0%)	2(14.3%)	
Male	78(66.7%)	4(40.0%)	11(57.9%)	31(66.0%)	11(73.3%)	9(75.0%)	12(85.7%)	
Underlying diseases								
Non	26(22.2%)	4(40.0%)	5(26.3%)	9(19.1%)	4(26.7%)	–	4(28.6%)	
diabetes	32(27.4%)	–	3(15.8%)	15(31.9%)	6(40.0%)	5(41.7%)	3(21.4%)	
Cardiovascular and cerebrovascular diseases (hypertension, cerebral infarction, cerebral hemorrhage, etc.)	70(59.8%)	6(60.0%)	10(52.6%)	31(66.0%)	7(46.7%)	11(91.7%)	5(35.7%)	
Kidney diseases (chronic renal failure, uremia, etc.)	16(13.7%)	1(1.0%)	3(15.8%)	4(8.5%)	3(20.0%)	4(33.3%)	1(7.1%)	
Cardiac diseases (atrial fibrillation, etc.)	16(13.7%)	–	3(15.8%)	4(8.5%)	5(33.3%)	3(25.0%)	1(7.1%)	
Liver diseases	6(5.1%)	–	–	2(4.3%)	1(6.7%)	2(16.7%)	1(7.1%)	
Psychiatric disorders, neurodegeneration	6(5.1%)	–	1(5.3%)	5(10.6%)	–	–	–	
Anemia (thrombocytopenia)	4(3.4%)	–	–	2(4.3%)	1(6.7%)	–	1(7.1%)	
Pulmonary diseases (tuberculosis)	10(8.5%)	–	2(10.5%)	1(2.1%)	2(11.8%)	3(25.0%)	2(14.3%)	
Others (hyperthyroidism, lymphadenoma, splenomegaly)	1(0.9%)	–	–	1(2.1%)	–	–	1(7.1%)	
Times from infection (hospitalization) to death (discharge), days (IQR)	18(1-82)	14(5-17)	15(3-36)	24(3-37)	24(1-53)	27(12-82)	11(2-28)	

### Morphological changes of peripheral blood smudge cells before and after bovine serum albumin treatment, and the discovery of neutrophil extracellular traps in neutral smudges

**Figure 1:**
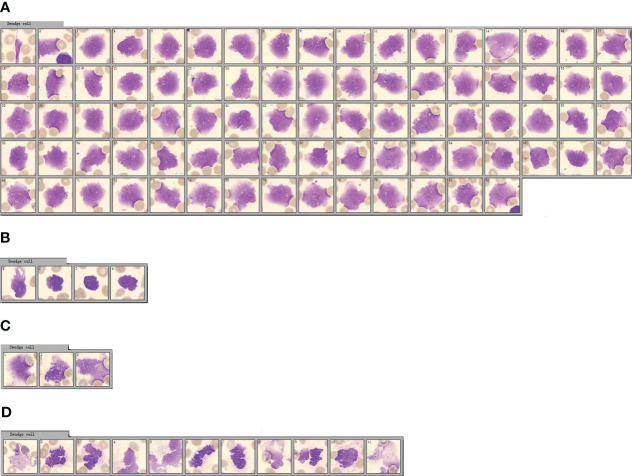
Morphological changes of peripheral blood smudge cells before and after bovine serum albumin (BSA) treatment. **(A)** Smudge cell count of the patient’s peripheral blood cell smear. (The serial numbers of pictures from left to right are 1-82). **(B)** Smudge cell count of peripheral blood cell smear of the blood sample treated with BSA. (The serial numbers of pictures from left to right are 1-4). **(C)** Smudge cell count on peripheral blood smear of patients with COVID-19. (The serial numbers of pictures from left to right are 1-3). **(D)** Smudge cell count on peripheral blood cell smears of patients with COVID-19 after the same blood sample was treated with BSA. (The serial numbers of pictures from left to right are 1-11).

The International Council for Standardization in Hematology (ICSH) recommends a solution for preparing and classifying smudge cells in peripheral blood after processing with one part of albumin and four parts of peripheral blood (25). Adding BSA to ethylenediaminetetraacetic acid (EDTA) acid-anticoagulated blood specimens for blood smears reduces the number of smudge cells and improves the consistency of morphological evaluation of peripheral blood smears in patients with chronic lymphocytic leukemia (CLL) patients (26, 27). In a routine blood sample from a patient with CLL, 82 smudge cells were observed when 100 white blood cells were counted using CellaVision DI-60 software (**Figure 1A**). However, when BSA was added to this CLL specimen and processed with peripheral blood at a ratio of 1:4, the total number of lymphatic cells was significantly reduced to only 4 when 100 white blood cells were counted using the CellaVision software (**Figure 1B**), indicating that BSA effectively protected the lymphocytes from disruption during the slide preparation process (26). Smudge cells have also been observed in blood samples from patients with COVID-19 (28). When 100 white blood cells were counted using CellaVision software in a routine blood sample from a COVID-19 patient, only three smudge cells were observed (**Figure 1C**). However, when serum albumin was added to the COVID-19 specimens and processed with peripheral blood at a ratio of 1:4, the total number of smudge cells did not decrease (**Figure 1D**).

From **Figures 1A, B**, it is evident that blood samples from patients with CLL treated with BSA had significantly fewer smeared cells. This verifies that BSA, as mentioned in the literature, can effectively protect lymphocytes from being destroyed during the smear process, preventing the formation of smeared cells. In **Figures 1C, D**, it is observed that the so-called “smudge cells” in the blood samples of patients with COVID-19 treated with BSA did not decrease. This confirmed that these cells were not smudge cells, but rather neutrophil nets. In contrast to eosinophils, basophils, and mast cells, neutrophils can be easily distinguished based on their morphology.

Smudge cells in peripheral blood smears are a characteristic of CLL (29). These smudge cells are degenerated lymphocytes are easily fragmented during slide preparation and appear as smudge cells under a microscope (30). The formation of smudge cells is related to the low expression of vimentin, which is associated with cell stiffness in lymphocytes and leads to lymphocyte fragility (31). The size, shape, and color of smudge cells in the blood smears of patients with COVID-19 are significantly different from those of lymphatic smudge cells, suggesting that smudge cells are neutral smudge cells. Neutrophils in patients with COVID-19 undergo cell death and their cell membranes are fragile and easily ruptured in smears (32). Detailed observation of COVID-19 smudge cells revealed segmented nuclei with swelling, granulations in the periphery of the swollen nucleus, and the appearance of filaments, which were significantly different from the characteristics of lymphocytic smudge cells. Fragments similar to those of the dyes were also observed (**Figure 1D**). Lymphatic smudge cells have unsegmented nuclei and no filamentous substances, and may contain naked nuclei. The equipment used in this experiment, CellaVision DI-60 software, did not perform detailed and accurate identification and classification of smudge cells.

### Morphological observation of NETs in blood smears from patients with COVID-19

**Figure 2:**
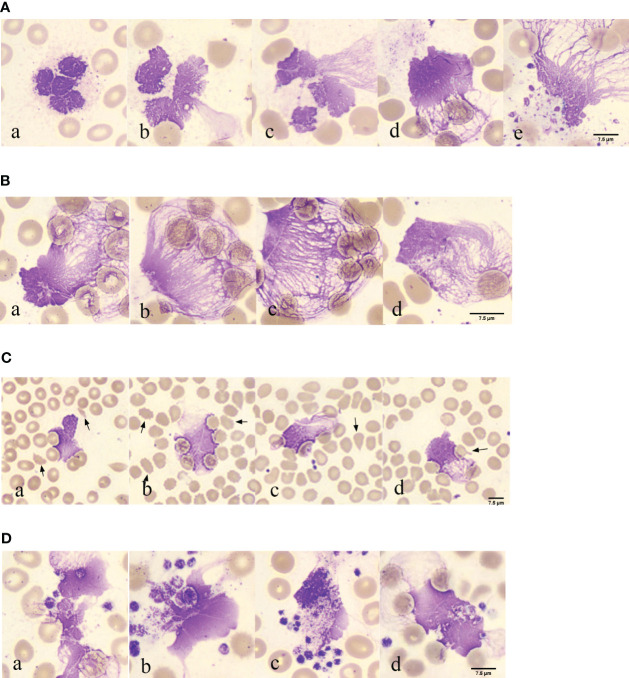
Morphological observation of neutrophil smudge NETs cells in patients with COVID-19 **(A)** Morphology of neutrophil smudge NETs cells in peripheral blood slide of patients with COVID-19 at different stages. **(B)** Erythrocyte is reticulated by neutrophil smudge cells NETs in peripheral blood slide of patients with COVID-19. **(C)** Poiki-locyte around neutrophil smudge cells NETs in p peripheral blood slide of patients with COVID-19. **(D)** Status of platelets around neutrophil smudge cells NETs in peripheral blood slide of patients with COVID-19.

In 117 blood smears from patients with COVID-19, neutrophils with lobulated nuclei and swollen cell bodies were observed in 117 blood smears from patients with COVID-19, with blurry and indistinct edges (**Figures 2A**, a). Neutrophils with exacerbated lobulation, accompanied by nuclear dissolution and fragmentation, showed unclear nuclear chromatin structures, light staining, and blurred nuclear contours, which began to erupt (**Figures 2A**, b). Neutrophils were found in the process of NETs eruption, with nuclear membrane rupture and disintegration, releasing fibrous chromosome-like material into the cytoplasm and causing destruction of the neutrophil structure (**Figures 2A**, c). Neutrophils were found to release a net-like structure, with red blood cells (black arrows) passing through the chromosome-like structure formed by their ejection and becoming trapped within the net-like structure (**Figures 2A**, d). Neutrophils with ruptured cell membranes, releasing granules, and forming net-like structures were also observed (**Figures 2A**, e). Furthermore, the phenomenon of neutrophil NETs net-like structures trapping red blood cells was observed, with an unclear neutrophil structure, swollen nucleus, and released net-like structure chemotactic to red blood cells (**Figures 2B**, a-d).

Abnormal red blood cells were found near the observable NETs (**Figure 2C**). Following ICSH guidelines for microscopy evaluation of fragmented cells, a higher proportion of fragmented red blood cells (≥1%) were found in patients with COVID-19 at different stages of the disease severity, which is unrelated to lung involvement and obvious intravascular coagulation (33). Patients with abnormal red blood cell morphology have lower peripheral blood oxygen saturation upon admission (34). Abnormal RBC morphology includes polychromasia, basophilic stippling, rouleaux formation, agglutination, spherocytes, fragmented cells, stomatocytes, nucleated RBCs, and mushroom cells (35), which are usually not associated with hemolysis. Some abnormal red blood cell shapes observed in patients with COVID-19 in peripheral blood smears upon admission included fragmented cells, spur cells, teardrop cells, and mushroom cells (**Figures 2C**, a-d). The fragmented cells were red or incompletely red cells with irregular sizes and shapes (**Figures 2C**, a). Spur cells had needle-like projections on the red cell surface with irregular spacing and varying lengths and widths (**Figures 2C**, b). The teardrop cells had a teardrop or pear shape (**Figures 2C**, c). Mushroom cells had a mushroom-like shape with variable sizes (**Figures 2C**, d). Spur cells may reflect early changes in RBC membrane components induced by SARS-CoV-2 infection, whereas stomatocytes may reflect a later-stage loss of elasticity. Spur cells may be damaged by the protein and lipid membrane components of red cells. The degree of abnormal RBC morphology correlates with disease severity, making peripheral blood smear a potential prognostic tool for patients with COVID-19 (34). The analysis of blood smears from patients with COVID-19-related anemia revealed several abnormal RBC shapes. The numbers of stomatocytes and bridge cells were very high, which is uncommon in other types of anemia blood smears. Recent research has suggested that RBC damage is a result of immune-mediated mechanisms and/or physical cell damage caused by COVID-19 microvascular changes. The observed biconcave shape of red blood cells in COVID-19 and complement activation may facilitate red blood cell aggregation and spontaneous agglutination and may promote typical microvascular thrombosis in COVID-19 (35).

Platelet aggregation was observed near the NETs (**Figure 2D**). In the EDTA-anticoagulated blood smear, platelets were randomly distributed or platelet aggregation was induced, with no platelet aggregation. Platelets around the neutrophil smudge cells were aggregated (**Figures 2D**, a). Normal platelets are two-sided, slightly convex discs, with a diameter of 2–4 μm, and larger in volume for newly formed platelets and smaller for mature ones. Blood smears were often scattered or clustered, mostly in circular, oval, or irregular shapes, and the cytoplasm was light blue or purple-red with small, evenly distributed purple-red granules in the central region. In this image, deeply stained platelets had increased volume, with a diameter greater than 4 μm, and the azure granules in the cytoplasm were small or fused into larger ones (**Figures 2D**, b). Platelets clustered around the neutrophils (**Figures 2D**, c). Red blood cell aggregation and platelets wrapping around neutrophil smudge cells were observed (**Figures 2D**, d). Platelet aggregation and the presence of giant platelets suggested increased platelet activity. These morphological features may be consistent with severe COVID-19, further confirming the important role of platelets in COVID-19 as a thrombotic complication (36). Several studies have shown that an increase in mean platelet volume (MPV) may reflect the risk of thrombosis and that MPV can be used as a marker of platelet activity in patients with pulmonary embolism. In a recent large-scale clinical evaluation, early cardiovascular events after COVID-19 were primarily caused by pulmonary embolism, atrial fibrillation, and venous thrombosis. Morphometric analysis using flow cytometry and electron microscopy showed that MPV, internal complexity, and the proportion of giant platelets increased in critically ill patients, confirming the role of platelets in COVID-19 cardiovascular complications (37).

In COVID-19, activated platelets may play a role in inducing NETs formation. In patients with COVID-19, the number of NETs increases with a significant decrease in lymphocytes. NETs density correlated with the severity of COVID-19. NETs also activate platelets via p-selectin, leading to platelet aggregation and thrombus formation (38). Another pathological mechanism associated with severe COVID-19 is an increase in platelet-neutrophil aggregates (39). The most common quantitative hematological abnormality in complete blood cell counts (CBC) is anemia, followed by an increase in neutrophils, a left shift of neutrophil nuclei, and a decrease in lymphocytes. The most significant morphological changes were chromatin aggregation in neutrophils, multiple abnormal nuclear shapes, and pseudo-Pelger-Huet anomalies. Lymphocytes exhibited abundant blue cytoplasm and/or lymphoplasmacytoid morphology, whereas monocytes showed abnormal shapes and vacuolization. The platelets can then aggregate. Red blood cells exhibit normocytic and normochromic features, with few nucleated red blood cells and rough granular eosinophils (40). The presence of fragmented blood cells is a morphological hallmark of thrombotic microangiopathy (TMA) in hemolytic anemia, with the main forms being helmet- and crescent-shaped (41).

Elevated D-dimer levels, prolonged prothrombin time, and decreased platelet counts reflect a close association between coagulation and COVID-19. Notably, despite the use of standard prophylactic anticoagulation with heparin, severe thrombotic complications may still occur (42). Analysis of platelet characteristics in COVID-19 revealed that these platelets have increased reactivity (increased aggregation and expression of P-selectin and CD40) and unique transcriptional characteristics of prethrombotic large and immature platelets. Platelet count, size, and maturity were associated with an increased risk of critical illness and all-cause mortality in hospitalized patients with COVID-19. Significant dysregulation of the coagulation cascade is observed in critically ill patients with COVID-19, including elevated D-dimer, fibrinogen, and von Willebrand factor. Thrombotic events, including pulmonary embolism, venous thrombosis, and ischemic stroke, are common in critically ill patients. A hypercoagulable state is a major pathological event in COVID-19, and thromboembolic complications are life-threatening. Platelets are the main effector cells for hemostasis and pathological thrombus formation (43). As the understanding of severe COVID-19 develops, hypercoagulability has become a core pathological feature and clinical complication. Thrombotic events are particularly common in critically ill patients with COVID-19, with an increased incidence of venous and arterial thromboembolism and even life-threatening complications such as pulmonary embolism, ischemic stroke, and myocardial infarction. Compared with bacterial pneumonia, SARS, or influenza pneumonia, COVID-19 deaths have increased microthrombi in the alveolar capillaries and a higher frequency of disseminated intravascular coagulation. Autopsy studies revealed small thrombi in the pulmonary arterioles in areas where the alveolar-capillary integrity was compromised (43).

### Compared to high fib and d-dimer, the nets/neutrophilic segmented granulocyte ratio is more easily assessable for predicting the risk of mortality in patients with COVID-19

**Figure 3:**
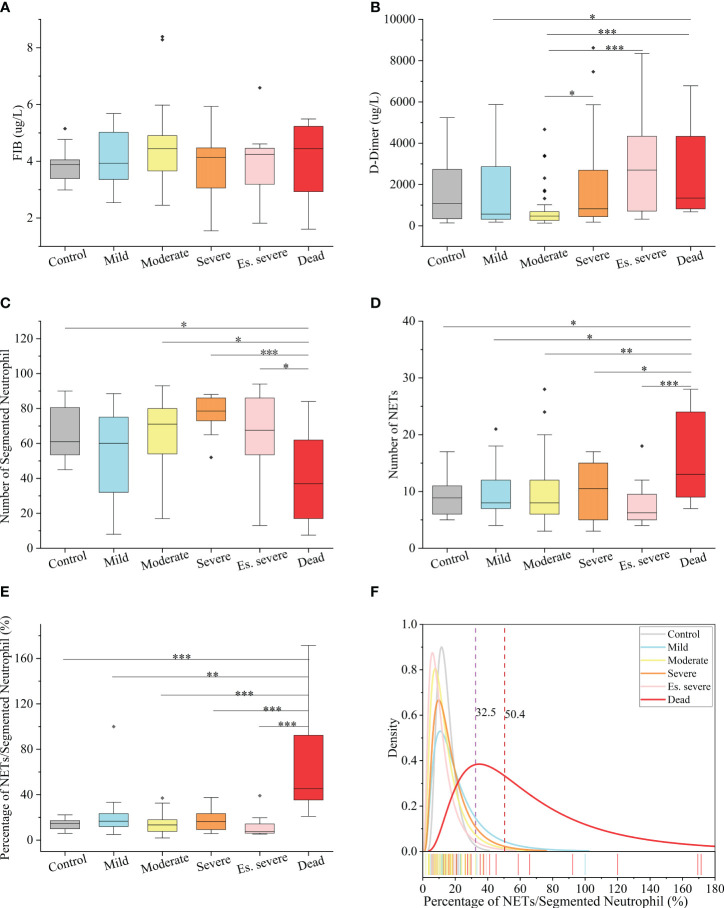
Hematological Index of COVID-19 (positive and negative) patients **(A, B)** Box and whisker (25–75 percentile) plots of FIB(g/L) and D-dimer(ug/L) in peripheral blood samples of patients with COVID-19 compared with the control group. **(C, D)** Box and whisker (25–75 percentile) plots indicating the count levels of segmented neutrophils and NETs in peripheral blood collected from samples infected with SARS-COV-2 viruses at specific times. **(E)** Box and whisker (25–75 percentile) plots depicting the divergence in each group by the defined index (NETs/Segmented Neutrophil (%)). **(F)** Density specific for each group visualized along the percentage (NETs/Segmented Neutrophil), highlighting the distinction of symptoms of patients with COVID-19. Dotted lines indicate the threshold of NNSR which divide reference interval on logarithmic normal analysis. The valid data range is mean ± 95%CI. ∗p < 0.05, ∗∗p < 0.01, ∗∗∗p < 0.001. Non-parametric multivariate ANOVAs with Dunn’s *post hoc* test for multiple comparisons.

In peripheral blood, FIB can be reported within the range of 0.35–10 g/L, and D-dimer can be reported within the range of 112–55845 µg/L. Notably, the FIB levels were not significantly different between the groups (**Figure 3A**), making it an unreliable predictor of disease progression. Similarly, the role of D-dimer as an early warning indicator of severe, critical, and fatal disease risk in adults remains unclear (**Figure 3B**). D-dimer (a degradation product of fibrinogen that indicates hypercoagulability) is a reliable marker of COVID-19 severity (44). The presence of coagulation disorders characterized by elevated D-dimer and FDP levels is strongly associated with more severe disease and higher mortality rates (45). Nonsurvivors exhibited higher D-dimer levels than survivors, with approximately 70% of nonsurvivors meeting the criteria for dispersive intrinsic coagulation during hospitalization, whereas the proportion was 0.6% for survivors. There is a positive correlation between the D-dimer levels and thrombus formation (46). Elevated D-dimer levels are the most common coagulation abnormality in COVID-19 (observed in up to 45% of patients) and are an independent risk factor for death (45). However, these findings do not agree with the results of our study. This discrepancy may be due to the lack of NETs observation methods in previous studies, coupled with the absence of quantitative analysis and statistics of NETs quantity in patients’ blood cells. However, the role of NETs in the coagulation system has been neglected in these studies. NNSR is a more significant risk factor for death than D-dimer level. Consequently, the estimation of risk factors for severe disease and death in these studies is questionable. Comprehensive descriptions of the clinical and virological courses pose challenges. Compared to the other groups, there was a significant decrease in the absolute count of neutrophilic segmented granulocytes (cells/100 cells) in the peripheral blood of the dead group (**Figure 3C**). Similarly, there was a significant decrease in the neutrophilic NETs count (cells/100 cells) in the peripheral blood of the dead group (**Figure 3D**). Additionally, there was a significant increase in NNSR in the peripheral blood of the deceased group (**Figure 3G**).

**Figure 4:**
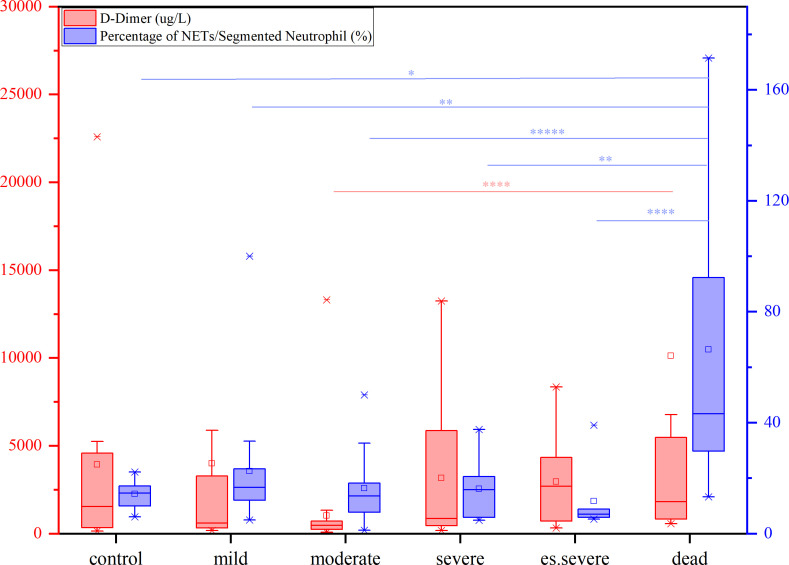
The contents of D-dimer (red) and percentage of NETs/Segment Neutrophil (purple) in patients with different symptoms (from mild to dead, compared with control group), nonparametric tests, *p<0.05, **p<0.01, ****p<0.0001.

The levels of FIB, a precursor of fibrin, were not significantly increased in patients with COVID-19. D-dimer, a degradation product of cross-linked fibrin, increased in acute thrombus. It also increased physiologically with age, cancer, infection, or other inflammatory states. Therefore, a negative D-dimer level helps exclude venous thromboembolism in patients with normal concentrations, but an elevated D-dimer level does not confirm venous thromboembolism (47). This conclusion is consistent with our results. Owing to the invasion of the body by the COVID-19 virus and a decrease in the body’s immune system, there was a significant decrease in the number of neutrophilic segmented granulocytes, especially in those at risk of death (**Figure 3C**). In the deceased group (indicators measured 3–4 days before death), the number of NETs was significantly increased (**Figure 3D**). In COVID-19 dead patients, although the number of NETs increased, NNSR also significantly increased (**Figures 3D, E**). **Figure 4** reveals that D-dimer exhibited a notable difference only in the moderate disease group (p < 0.0001), rendering it inadequate for predicting the risk of death in patients with COVID-19. In contrast, the NETs/segment neutrophil index demonstrated a substantial increase (more than two times) in the death group, making it a more effective predictor of mortality risk in patients with COVID-19 (**Figure 4**). The serum levels of NETs in many hospitalized patients with COVID-19 have increased. Severe COVID-19 appears to be defined by an increase in neutrophils, IL-1β, IL-6, and D-dimer, suggesting an activated coagulation system (4). The number of neutrophils in the circulation of patients increases, and patients with severe COVID-19 exhibit high levels of NETs markers in their circulation. The higher levels of NETs in the circulation of patients with COVID-19 may be due to an increase in the neutrophil count during the severe stage and, more importantly, due to an increase in the ability of neutrophils to release NETs (46).

Other studies have also reported the lethal mechanism of intrinsic coagulation (NETs) caused by the COVID-19 virus. Due to the activation of the intrinsic coagulation pathway, neutrophils are decorated with platelets in COVID-19, especially in severe cases, and form cell aggregates. Removing neutrophils from the cells of patients with COVID-19 resulted in a depleted phenotype that reduces the spontaneous oxidative burst in response to lipopolysaccharide (LPS). Activated neutrophils are more prone to aggregation and the formation of high-density NETs, which can block tubular structures. However, under inflammatory conditions, the local degradation of stable NETs may be excessive, resulting in vascular occlusion. Neutrophil activation is an important component of the immune pathogenesis of COVID-19. Many pulmonary microvessels are blocked by neutrophil aggregates or neutrophil-derived DNA (48). This has led to an increasing trend in the ratio of NETs/neutrophilic segmented granulocytes in patients with COVID-19. When the NETs ratio reaches a high value, it can increase the risk of thrombotic death. From a 95% confidence perspective, a NETs ratio > 50% increases the risk of thrombus death. The increase in the NETs ratio was mainly due to a decrease in the number of segmented neutrophils. Abnormal coagulation parameters (elevated D-dimer and FDP levels) are associated with disease severity. Although free DNA is not a highly specific marker for NETs, it is closely related to the absolute neutrophil count. Elevated levels of D-dimer and cell-free DNA have also been reported (47). The number of neutrophils in circulation increased. Excess NETs can cause tissue damage and harm to the host. This goes beyond their direct toxic effects on tissues, but when released into the circulation, they lead to inappropriate activation of coagulation and thrombus formation, blocking blood flow and oxygen, as well as the supply of nutrients to tissues. During COVID-19, ~60% of patients with severe disease develop COVID-19-associated coagulopathy (CAC), which is characterized by elevated levels of D-dimer in the circulation, unchanged or decreased platelet count, shortened prothrombin time, and increased risk of thrombosis (46). However, the aforementioned literature does not provide experimental data on NETs.

## Discussion

Compared to traditional methods of detection, utilizing blood cell morphology to observe and count the morphology of NETs in peripheral blood slides and subsequently calculating the ratio of NETs to neutrophilic segmented granulocytes to assess the risk of patients with COVID-19 is a simple, direct, fast, and economical method with good clinical value.

The introduction of two indices, namely the quantity of NETs (number per 100 cells) and the ratio of NETs to neutrophilic segmented granulocytes (NNSR), allows for a quantitative description of the changes in NETs in peripheral blood. Based on the definition of the ratio of NETs to neutrophils, low-risk and high-risk zones can be established as new criteria for assessing COVID-19 mortality risk. An NNSR between 32.5% and 50.4% was considered a suspicious indicator and required further testing. An NNSR greater than 50.4% indicated a high risk of mortality (**Figure 3F**).

The peripheral blood of patients with COVID-19 provides a good scenario for observing NETs. The formation process, count, and ratio changes of neutrophil NETs can be observed on blood slides, providing an effective monitoring tool for disease prognosis. Furthermore, NET-related parameters can be used as monitoring and risk assessment indicators during the treatment of diseases, such as viral infections, tumors, and microbial infections.

Thrombosis, the local clotting of blood, can occur in arterial or venous circulation and pose a significant medical risks (49–51). Acute arterial thrombosis is the main cause of myocardial infarction and stroke, whereas acute venous thrombosis is the primary cause of pulmonary embolism. Myocardial infarction, stroke, and pulmonary embolism pose severe threats to the lives of affected patients (52–54). The mechanisms underlying the pathological changes in blood vessel walls and blood that lead to thrombosis are not yet fully understood. Therefore, developing safer and more effective antithrombotic drugs is of great scientific and clinical significance (55, 56). SARS-CoV-2 infection triggers the activation of monocytes, which along with monocyte-derived microvesicles and tissue factors expressed by endothelial cells activate the extrinsic coagulation pathway, leading to fibrin deposition and blood clotting (57, 58). Neutrophils release NETs that activate the intrinsic coagulation pathway, bind to and activate platelets, and amplify blood clotting (8). However, this model does not further elaborate on the weighted impact of intrinsic and extrinsic coagulation on patient mortality caused by COVID-19-induced thrombosis. FIB and D-dimer can be easily quantified through blood tests; however, the difficulty in quantifying NETs is a major reason why they cannot be discussed in depth. This model does not indicate the weighted impact of intrinsic and extrinsic coagulation on the mechanism of thrombus formation nor does it indicate which type of clotting has a greater impact on patients.

NETs play important roles in the pathogenesis of COVID-19-associated arterial thrombosis (59–61). Neutrophil extracellular traps (NETs) are formed when neutrophilic intracellular proteins are degraded by elastase (NE), causing nuclear disintegration. Peptidylarginine deiminase 4 (PAD4) promotes histone depolymerization and facilitates chromosomal DNA release. Gasdermin D produces pores in the cell membrane, promoting cell membrane rupture and discharge of DNA and its associated molecules, resulting in the formation of NETs (44, 62). Histones H3 and H4 in NETs activate the intrinsic coagulation pathway by interacting with FXI and XII and downregulating thrombomodulin, leading to a procoagulant state. Endothelial injury activates the extrinsic pathway by expressing TFIII, which binds to FVII and triggers the coagulation cascade (63, 64). The interaction of thrombin, FXa, and the TFIII-FVII complex with protease-activated receptors (PARs) leads to platelet activation and aggregation, followed by the release of granule contents such as P-selectin (65). Excessive circulating NETs can trigger an inflammatory cascade, leading to tissue damage, small vessel occlusion, and microthrombus formation in the lungs and cardiovascular and renal systems. This can lead to permanent damage (5 to 66). The mechanism is thought to be caused by abnormal signaling during the cytokine storm, where NETs induce macrophages to secrete IL-1β, further promoting the formation of NETs (67).The impact on the coagulation system is associated with significant incidence and mortality rates (68, 69). Coagulation disorders can lead to arterial and venous thrombosis, particularly pulmonary embolisms and microthrombi. The incidences of thrombus formation and thrombus-related complications are high in adults with severe COVID-19 (70). Microthrombus not only exist in pulmonary vessels but also in other organs, and acute limb ischemia (ALI) is a severe complication of COVID-19 (47, 71). The interaction between NETs and activated platelets plays a role and enhances procoagulant activity in patients with acute stroke and carotid artery occlusion (72). Neutrophil depletion reduced blood-brain barrier (BBB) breakdown at 14 days and promoted neovascularization. It has also been observed that the percentage of circulating neutrophils is higher in the peripheral blood 3 days after stroke, as determined by flow cytometry for blood cell counts. Neutrophils cause delayed vascular damage. Stroke causes neutrophil accumulation in the brain (73). Immune cell control of thrombus formation has cell-type specificity that is limited to neutrophils. Neutrophils drive myocardial infarction thrombosis formation (74). This highlights the importance of effective thromboprophylaxis and treatment of patients with COVID-19 with thrombus complications. Given the established association between NETs and thrombus formation in many inflammatory diseases, these data suggest that the role of NETs in COVID-19-related thrombus formation warrants systematic and prospective investigations.

**Figure 5:**
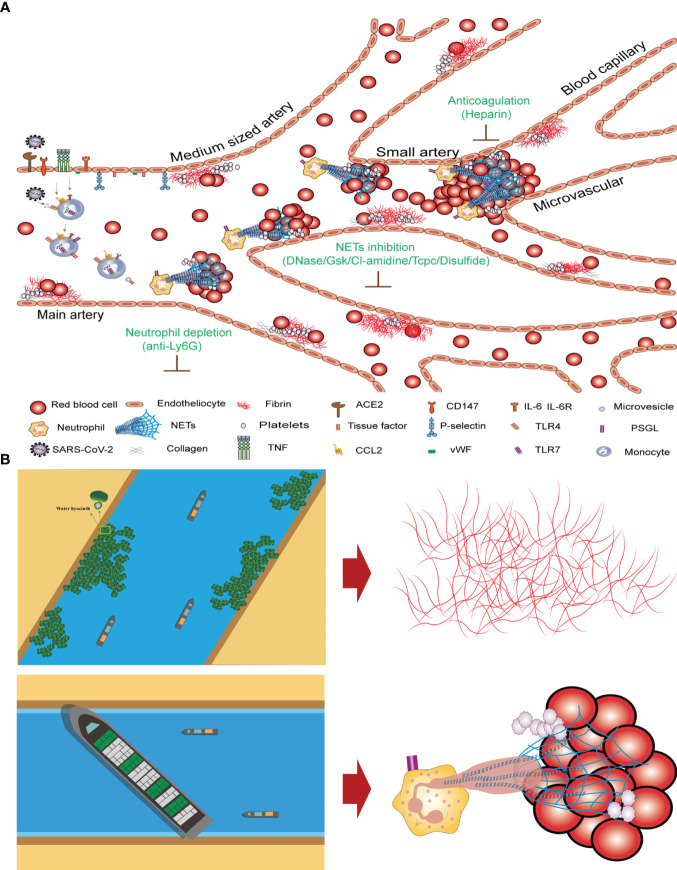
Mechanism underlying COVID-19 thrombosis and the difference between intrinsic and extrinsic coagulation **(A)** Thrombosis mechanism of intrinsic and extrinsic coagulation in new crown patients. **(B)** Differences in coagulation mechanisms of FIB and NETs+RBC complex.

The clotting mechanisms of fibrinogen and NETs differ significantly. Thrombin cleaves fibrinogen into fibrin monomers, which are then crosslinked to form stable fibrin clots. The concentration of fibrinogen in human plasma is approximately 3 g/L. Fibrinogens are highly insoluble protein polymers with needlelike crystal structures. Both FIB and D-dimer levels are indicators of fibrin clotting. Our study revealed no significant correlation between high FIB concentrations and COVID-19 mortality (**Figure 3**). After neutrophil NETs formation, the complex of NETs and red blood cells forms with a diameter of about 40–70 µm, which under the influence of blood flow can block arteries and form blood clots (**Figure 5A**). The fibrinogen-independent clotting mechanism is similar to that of water hyacinths in waterways, which do not cause fatal blockages in waterways or obstruction of blood vessels. However, the NETs+RBC complex resembled a large vessel in a waterway that could obstruct the waterway, similarly blocking blood vessels (**Figure 5B**). In **Figure 5**, we can observe nets enmeshing several red blood cells (Red blood cell diameter= 7.0–7.6 µm) and the formation of emboli in the micro and small vessels (Microvessels diameter = 4–6 µm). The count of NETs in the peripheral blood of patients with COVID-19 shows that the ratio of NETs to neutrophilic segmented granulocytes is greater than 85%, which increases the risk of vascular obstruction and easily leads to patient death. This ratio can serve as a warning indicator of the COVID-19 mortality risk.

**Table 2** shows that drugs targeting NETs to induce internal coagulation have not been the focus of COVID-19 drug development.

**Table 2:** Potential thrombosis-related therapeutics for COVID-19.

	Drugs type	Drugs name	Mechanism of action
Antiplatelet drugs	TXA2 inhibitors	Aspirin	Inhibit COX
	ADP P2Y12 receptor antagonist	Thiopyridines, such as clopidogrel	Irreversibly inhibit platelet ADP receptors
		Non-thienopyridines, such as ticagrelor	Directly and reversibly inhibit platelet P2Y12 receptors
	GP lI b/IIIa receptor inhibitors	Abximab	The steric hindrance was used to prevent the ligand from binding to GPIIb/IIIa
		Tirofiban	Specific non-peptide GPIIb/IIIa receptor antagonists mimic GPIIb/IIIa receptors to recognize RGD peptides
	Phosphodiesterase inhibitors	Dip	Phosphodiesterase-5 inhibitors
		Cilostazole	Phosphodiesterase-3 inhibitors
Anticoagulant drugs	Vitamin K antagonists	Warfarin	Inhibit liver synthesis of coagulation factors II, VII, IX, X
		LMWH	Enhance the activity of antithrombin and stimulate the release of TFPI from vascular endothelial cells
	Non-vitamin K antagonists	Dabigatran	Inhibition of coagulation factor IIa
		Rivaroxaban	Inhibition of FXa
Fibrinolytic drugs	Nonspecific plasminogen activator	Streptokinase, urokinase	Non-specific activation of plasminogen
	Specific plasminogen activator	Rt-PA	Selective activation of plasminogen
NETs degrading drugs		Anti-Ly6G	Promote neuter depletion
		DNase 1	Degraded DNA
		Cl-amidine	Inhibition of PAD4
		TcpC	Inhibition of PAD4
		GSK	Inhibition of PAD4
		Disulfide	GSDMD

TXA2, Thromboxen A2; COX, Cyclooxygenase; ADP, Adenosine diphosphate; GP, Glycoprotein; RGD, Arginine-Glycine-Aspartate; Dip, Dipyridamole; LMWH, Low molecular weight heparin; TFPI, Tissue factor pathway inhibitor; DNase 1, Deoxyribonuclease 1.

**Table 2** shows the drugs possibly related to thrombosis treatment. Antiviral therapy is an effective treatment for COVID-19. Antiviral drugs, such as the small-molecule antiviral drug nirmatrelvir/ritonavir (Paxlovid) (75, 76), which have been marketed since the outbreak, can only reduce the likelihood of developing severe cases. It is indicated for the treatment of mild-to-moderate COVID-19 in adults with high-risk factors that progress to severe disease.

In terms of antiplatelet therapy drugs, dipyridamole belongs to the phosphodiesterase inhibitor class and can inhibit platelet aggregation by increasing the intracellular cyclic adenosine monophosphate concentration. Dipyridamole has a specific affinity for SARS-CoV-2 protease Mpro and inhibits SARS-CoV-2 replication *in vitro*. Patients treated with dipyridamole had decreased D-dimer levels, improved platelet and lymphocyte counts, and a trend towards improved clinical cure and discharge rates compared with the control group (**Table 2**, row 7).

In terms of anticoagulant therapy, owing to the obvious hypercoagulable state of patients with COVID-19, especially those with severe or critical illness, and factors such as prolonged bed rest and steroid therapy, the incidence of thrombotic events is higher (77), which severely affects patient prognosis. Both domestic and international guidelines and expert consensus recommend that all hospitalized patients with COVID-19 without contraindications should consider using prophylactic doses of low molecular weight heparin (LMWH) (78, 79). Currently, the clinical treatment of COVID-19-induced thrombosis mainly relies on LMWH as an anticoagulant. LMWH can inhibit the activation of thrombin and subsequent fibrin generation, and reduce inflammation. Unfortunately, clinical results have not shown consistent efficacy, and anticoagulants have not been able to prevent thrombosis. Additionally, anticoagulants were not very effective in clearing pre-existing blood clots (**Table 2**, row 10).

Thrombolytic drugs are a potential treatment for COVID-19 thrombotic complications (80, 81) and ARDS (82). In China, drugs commonly used for thrombolytic therapy include urokinase, streptokinase, and rt-PA, which promote fibrinolysis by activating plasminogen (**Table 2**, row 13-15).

In terms of targeted therapy for NETs, the important role of neutrophils in the pathophysiology of COVID-19-related thrombosis makes them highly scrutinized targets (**Figure 5A**). For example, the use of anti-Ly6G antibodies to clear neutrophils can reduce NETs production in neutrophils. NETs are composed of DNA as a backbone embedded with histones (primarily citrullinated histone 3, CitH3), neutrophil elastase (NE), myeloperoxidase (MPO), antimicrobial peptides (LL-37), and serine proteases. DNase1 can promote NETs degradation (83). Research results have shown that pharmacological inhibition of GSDMD by disulfide can prevent NETs release (84) (**Table 2**, row 16-21) (**Figure 5A**). Targeting NETs with anti-thrombotic drugs could bring hope to patients, as severe complications such as myocardial infarction, stroke, and pulmonary embolisms caused by thrombi can lead to rapid death.

In the ongoing combat against COVID-19 and its constant evolution, developing targeted drugs for NETs is a future research direction.

## Data availability statement

The original contributions presented in the study are included in the article/Supplementary Material, further inquiries can be directed to the corresponding author/s.

## Ethics statement

Ethical approval was not required for the studies involving humans because due to the use of the patient’s routine monitoring of the remaining blood, there is no additional risk of unsuitable for the patient. Exemption from informed consent will not adversely affect the rights and health of the subjects. This study does not use the medical records and specimens that the subjects have explicitly refused to use before. The studies were conducted in accordance with the local legislation and institutional requirements. The human samples used in this study were acquired from the cases/biological specimens obtained from previous clinical diagnosis. Written informed consent to participate in this study was not required from the participants or the participants’ legal guardians/next of kin in accordance with the national legislation and the institutional requirements.

## Author contributions

QW: Funding acquisition, Writing – original draft, Writing – review & editing. YQ: Writing – original draft, Writing – review & editing. JM: Writing – original draft. KZ: Software, Writing – original draft. GX: Methodology, Writing – original draft. YL: Data curation, Writing – original draft. LX: Software, Writing – original draft. RA: Writing – review & editing. QL: Writing – original draft. XH: Writing – original draft. JZ: Writing – review & editing. HY: Writing – original draft, Writing – review & editing.

## Glossary

NETs: neutrophil extracellular traps
FIB: fibrinogen
DD: D-dimer
NNSR: NETs/neutrophilic segmented granulocyte ratio
SARS-CoV-2: severe acute respiratory syndrome coronavirus 2
TF: tissue factor
PAMPs: pathogen-associated molecular patterns
PSGL: P-selectin glycoprotein ligand
VWF: von willebrand factor
TFPI: tissue factor pathway inhibitor
PAI 1: plasminogen activator inhibitor 1
